# Atypical and Severe Psychotic Presentations in Adults with Autism Spectrum Disorder: Clinical Insights from Three Cases

**DOI:** 10.1192/j.eurpsy.2026.11081

**Published:** 2026-07-31

**Authors:** A. K. Rios-Landeo, S. Albarzani, E. Ros-Cucurull, F. Palma-Alvarez, O. Akpubi

**Affiliations:** 1 Psychiatry, University Hospital Waterford - HSE, Waterford, Ireland; 2 Psiquiatria, Hospital Universitari Vall d’Hebron, Barcelona, Spain

## Abstract

**Introduction:**

Autism Spectrum Disorder (ASD) is recognised as a significant vulnerability factor for the development of psychotic disorders in adulthood. Presentations are frequently atypical and variable trajectories that challenge timely recognition and management. Overlapping features can obscure emerging psychosis. Pharmacological treatment is often constrained by poor tolerability, inconsistent efficacy, and higher rates of treatment resistance compared with non-ASD populations. Despite these clinical challenges, systematic case-based data in adults remain limited, hindering the development of tailored therapeutic approaches.

**Objectives:**

To present three adult cases with ASD exhibiting atypical or severe psychotic symptoms, highlighting diagnostic complexity, functional impact, and treatment challenges.

**Methods:**

Three adults with ASD were systematically evaluated through comprehensive clinical histories, structured psychiatric assessments, detailed mental state examinations, and longitudinal treatment records. Emphasis was placed on atypical psychotic features, functional impairment, and individualized pharmacological and psychosocial management strategies.

**Results:**

**Table 1.** Table 1. Long description.

Case	Age	Sex	Primary Diagnosis	ASD Features	Psychotic/Affective Symptoms	Triggers	Treatment	Outcome
1	25	M	Schizoaffective	Social withdrawal, rigid behaviours	Severe negative symptoms, self-harm, minimal insight	Psychosocial stressors	Clozapine + Aripiprazole + Valproate	Partial remission, ongoing support (residential care)
2	30	F	Schizoaffective	Social naivety, sensory sensitivity	Polymorphic delusions, affective dysregulation	Psychosocial stressors, sleep loss	Lithium + Quetiapine + Paliperidone + Psychotherapy + psychosocial support	Full remission
3	32	F	Delusional Disorder	High-functioning ASD, social naivety, rigid behaviours	Persecutory delusions, severe self-harm, minimal insight	Psychosocial stressors, sleep loss	Olanzapine + Aripiprazol + psychosocial support	Full remission

**Conclusions:**

Psychosis in ASD is not only more frequent than previously recognised but also clinically distinct, often presenting with polymorphic, severe, and treatment-resistant profiles. The three cases illustrate key clinical scenarios: (1) profound negative symptoms hindering recovery, (2) polymorphic psychosis complicated by pharmacological intolerance, and (3) severe persecutory delusions masked by high cognitive functioning and minimal insight. These insights stress the need for early recognition, tailored pharmacological strategies, structured psychosocial interventions, and sustained multidisciplinary care. Broader clinical awareness and targeted research are essential to improve outcomes in this highly vulnerable subgroup.

(Beebani et al., 2022; Deste et al., 2020; Strålin & Hetta, 2019; Yalcin et al., 2016; Bargiela et al., 2016; Downs et al., 2017; Chisholm et al., 2015; Sporn et al., 2004)

**Disclosure of Interest:**

None Declared

## Long descriptions

### Table 1. Long description

The table consists of nine columns: Case, Age, Sex, Primary Diagnosis, A S D Features, Psychotic/Affective Symptoms, Triggers, Treatment, and Outcome.

* Case 1: A 25-year-old male diagnosed with Schizoaffective disorder. Features include social withdrawal and rigid behaviors. Symptoms include severe negative symptoms, self-harm, and minimal insight. Triggered by psychosocial stressors. Treated with Clozapine plus Aripiprazole plus Valproate. Outcome is partial remission with ongoing residential care support.

* Case 2: A 30-year-old female diagnosed with Schizoaffective disorder. Features include social naivety and sensory sensitivity. Symptoms include polymorphic delusions and affective dysregulation. Triggered by psychosocial stressors and sleep loss. Treated with Lithium plus Quetiapine plus Paliperidone plus Psychotherapy plus psychosocial support. Outcome is full remission.

* Case 3: A 32-year-old female diagnosed with Delusional Disorder. Features include high-functioning A S D, social naivety, and rigid behaviors. Symptoms include persecutory delusions, severe self-harm, and minimal insight. Triggered by psychosocial stressors and sleep loss. Treated with Olanzapine plus Aripiprazol plus psychosocial support. Outcome is full remission.

Navigate back to Table 1..

